# Epigenetic changes in poultry due to reprogramming of the gut microbiota

**DOI:** 10.1093/af/vfab063

**Published:** 2021-12-17

**Authors:** Aleksandra Dunislawska, Anna Slawinska, Maria Siwek, Marek Bednarczyk

**Affiliations:** Department of Animal Biotechnology and Genetics, Bydgoszcz University of Science and Technology, 85-084 Bydgoszcz, Poland

**Keywords:** bioactive substances, chicken, DNA methylation, in ovo stimulation, intestinal microbiota, miRNA activity

**Implications**

- Expression of many genes was downregulated in the spleen and liver after in ovo stimulation with prebiotics and synbiotics.
- In ovo stimulation with bioactive substances on day 12 of egg incubation activates epigenetic mechanisms (i.e. DNA methylation and microRNA (miRNA) expression).
- In ovo administration of prebiotics and synbiotics affects changes in the level of DNA methylation, which depends on the chicken tissue and genotype.
- In ovo delivery of probiotics or synbiotics has a significant impact on the expression of miRNAs.
- The transgenerational effects are still undetermined.

## Introduction 

In poultry production, genetic and phenotypic factors are taken into account when selecting for the new traits. But epigenetics actually controls how the genetic make-up of the animal is used. Epigenetics is driven by environmental cues that activate or deactivate various mechanisms controlling gene expression on transcription, post-transcription, and translation levels. Epigenetics studies the changes inherited during mitosis that affect the expression of genes that do not mediate modifications to the DNA sequence. This regulation plays a key role in the development and differentiation of body cells (Egger et al., 2004). Numerous scientific reports indicate that modulation of the intestinal environment has a significant impact on the regulation of epigenetic mechanisms in animals (Sharma et al., 2020), which is an area for research in the field of the influence of microbiome–host interactions on the modulation of gene expression. To date, we know that the intestinal microbiota takes part in metabolism, immunomodulation, and neurological function (Zheng et al., 2020). In the course of an animal’s life, microbes first colonized the neonatal gut, provided antigens for immune system maturation, and went on to protect the gut ecosystem while producing various metabolites. What differences in growth and health outcomes might be uncovered if we reprogram microbiota early enough to actually pinpoint epigenetic changes? This line of study was possible due to the development of in ovo technology in poultry (Siwek et al., 2018). Of the many different applications of in ovo technology, the potential to intervene within the embryonic microbiota composition by delivering bioactive compounds such as prebiotics, probiotics, and synbiotics on day 12 of egg incubation was particularly interesting. There are many beneficial phenotypic effects of such in ovo stimulation, including changes to intestinal health, meat quality, and immune system development. The aim of this review was to present the available scientific data on the epigenetic regulation of gene expression under the influence of changes in the intestinal microbiota caused by in ovo stimulation on day 12 of egg incubation.

## Intestinal Microbiota of Poultry

Intestinal microbiota is a complex population of microorganisms inhabiting intestinal walls and lumen of the gastrointestinal tract (**GIT**) (Rolhion and Chassaing, 2016). The habitats within the GIT are very diverse, providing different environments not only between particular intestinal segments, but even also within mucosal and luminal sites of the same segment. As a consequence, the microbial communities differ between the segments (from crop to cloaca) and sampling sites (mucosal vs. luminal content). In chickens, the GIT is relatively short, which results in a fast transit time of the digesta through the intestines. For this reason, the proximal intestinal segments are not colonized by very abundant and diverse microbiota (e.g., crop 10^3^ to 10^4^ CFU/g; *Lactobacilli* and *Streptococci*), in contrast to ceca, which is the most predominant niche for intestinal microbiota in chickens (10^11^ to 10^12^ CFU/g; *Ruminococci*, *Bacteroides*, *Clostridia*, *Streptococci*, *Enterococci*, *Lactobacilli*, and *E. coli*) (Yadav and Jha, 2019). 

**Figure 1.**
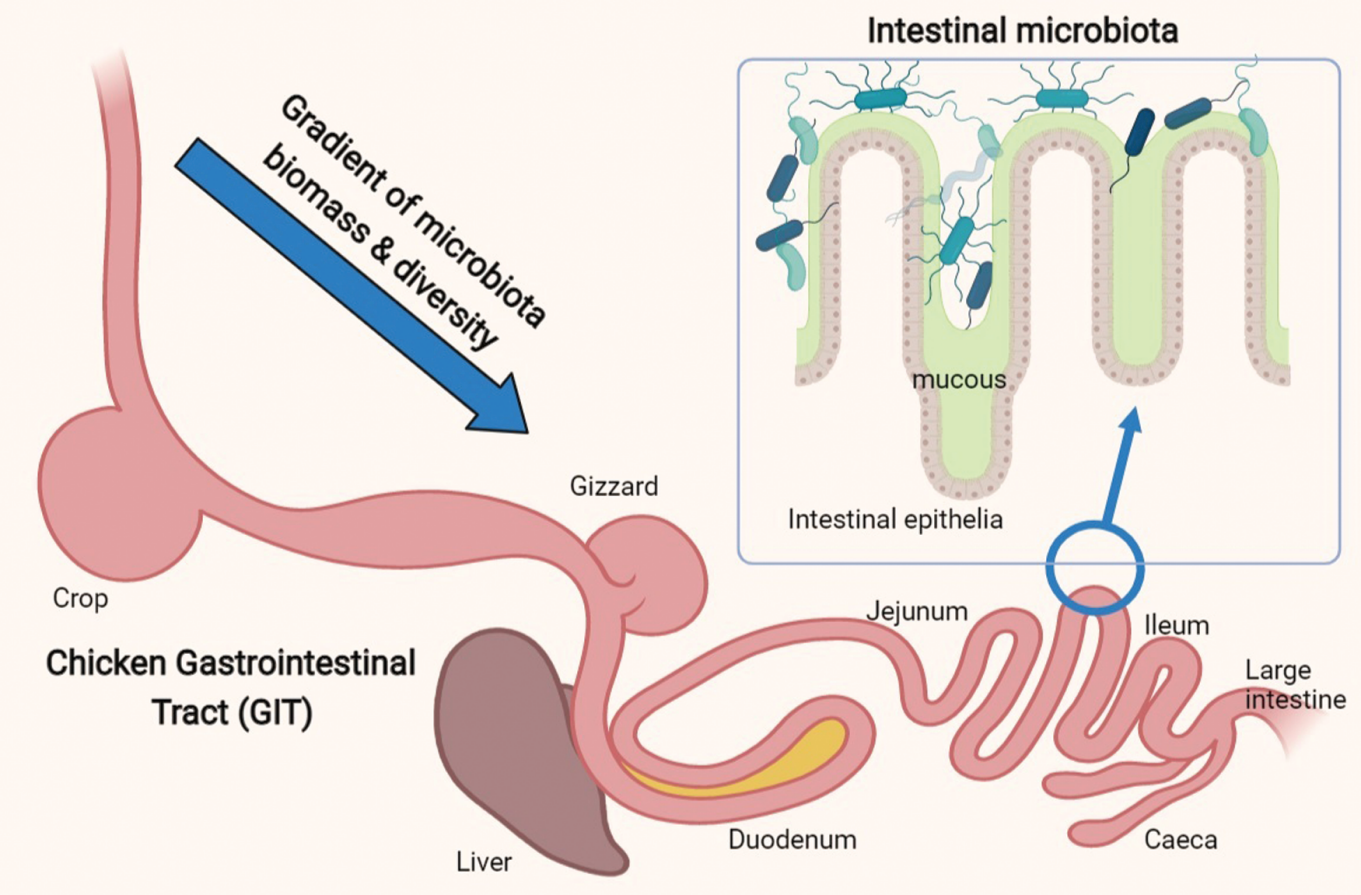
Schematic diagram of the chicken GIT and the epithelia colonized by intestinal microbiota (created in BioRender.com).

Intestinal microbiota contributes to the host’s gut homeostasis, health, immune status, and metabolism. The interplay between intestinal microbiota and the immune system is important at the early stages of the immune system development. Gut bacteria provide necessary stimuli to train the neonate’s innate and adaptive immune system, so that the adult individuals respond more effectively to infectious and inflammatory diseases later in life (Zheng et al., 2020). Commensal bacteria colonizing the mucosal epithelia create a protection from the pathogenic strains. The healthy microbiota is resilient to changes and, therefore, suppresses the growth of pathogens by competitive exclusion (Figure 1). There are different mechanisms of the competitive exclusion, such as passive competition for ecological niche (i.e., GIT mucosa) or nutrients, or active elimination of the competing bacteria by secreting toxins and antimicrobials (Bauer et al., 2018).

Another function of the intestinal microbiota is to take part in fermentation of nutrients not directly available to the host. This way, the gut microbiota not only participates in digestion but also delivers metabolites, which are important in the host’s metabolism. An important group of metabolites are short-chain fatty acids (**SCFA**s), which attribute to approximately 10% of the carbon source for the host and are also important metabolism mediators (e.g., butyrate). The metabolites produced by the intestinal microbiota are absorbed by the intestinal epithelia and reach the liver via the portal vein. The connection between the metabolites produced by intestinal microbiota and the host’s metabolic system is called the “gut–liver axis.” As such, the intestinal microbiota influences the chicken’s metabolism and productivity. Diaz Carrasco et al. (2019) reviewed the relationship between intestinal microbiota and performance in broiler chickens, expressed by growth and feed efficiency. Although there might be a correlation between microbial diversity and high- and low-productivity values, metabolism seems to depend on the particular taxa found in different segments of the GIT rather than the overall diversity.

The composition of the microbiota is not fixed, even though there is a genetic component to it and a certain level of resilience. Still, the diversity and the taxonomic composition of the intestinal microbiota can be manipulated with the environmental factors. A group of the most powerful microbiota modulators are antibiotics, which used to be applied in poultry as growth promoters but are now banned in many countries due to the risks of inducing antimicrobial resistance (Yadav and Jha, 2019). There are many more sustainable ways to modulate the intestinal microbiota so that it exerts beneficial effects (or the most desirable effects) in the host. These modulatory factors are associated with fiber-rich diets and dietary supplements, such as prebiotics, probiotics, and synbiotics, as well as various feed additives including enzymes, organic acids, natural extracts, essential oils, and other “functional foods” (Kogut, 2019; Yadav and Jha, 2019). Other approaches to modulate intestinal microbiota are to use fecal transplants or microbiota-based metabolite therapy, microbiota engineering, or bacteriophages; however, those are still less explored areas (Kogut, 2019). Whichever method of microbiota modulation is used, the window of opportunity of effectively colonizing the gut with the desired microbiota is limited. Therefore, in ovo technology is one of the most interesting approaches to deliver beneficial stimuli before other confounding environmental factors.

## In Ovo Stimulation Strategy

In a natural setting, the inoculation of the neonate chicks with the maternal microbiota is done at hatching. A hen’s microbiota is present on the eggshell and in the litter. The young chicks are exposed to these microorganisms during hatching and receive the first microbiota inoculation. Modern hatching technology is based on automated processes and almost maintenance-free incubators. Lack of contact with the hen, and sterilizing the eggs, deprives the neonatal chicks of maternal microbiota. But, at the same time, the technological development allows for an early intervention, which is called in ovo technology.

**Figure 2.**
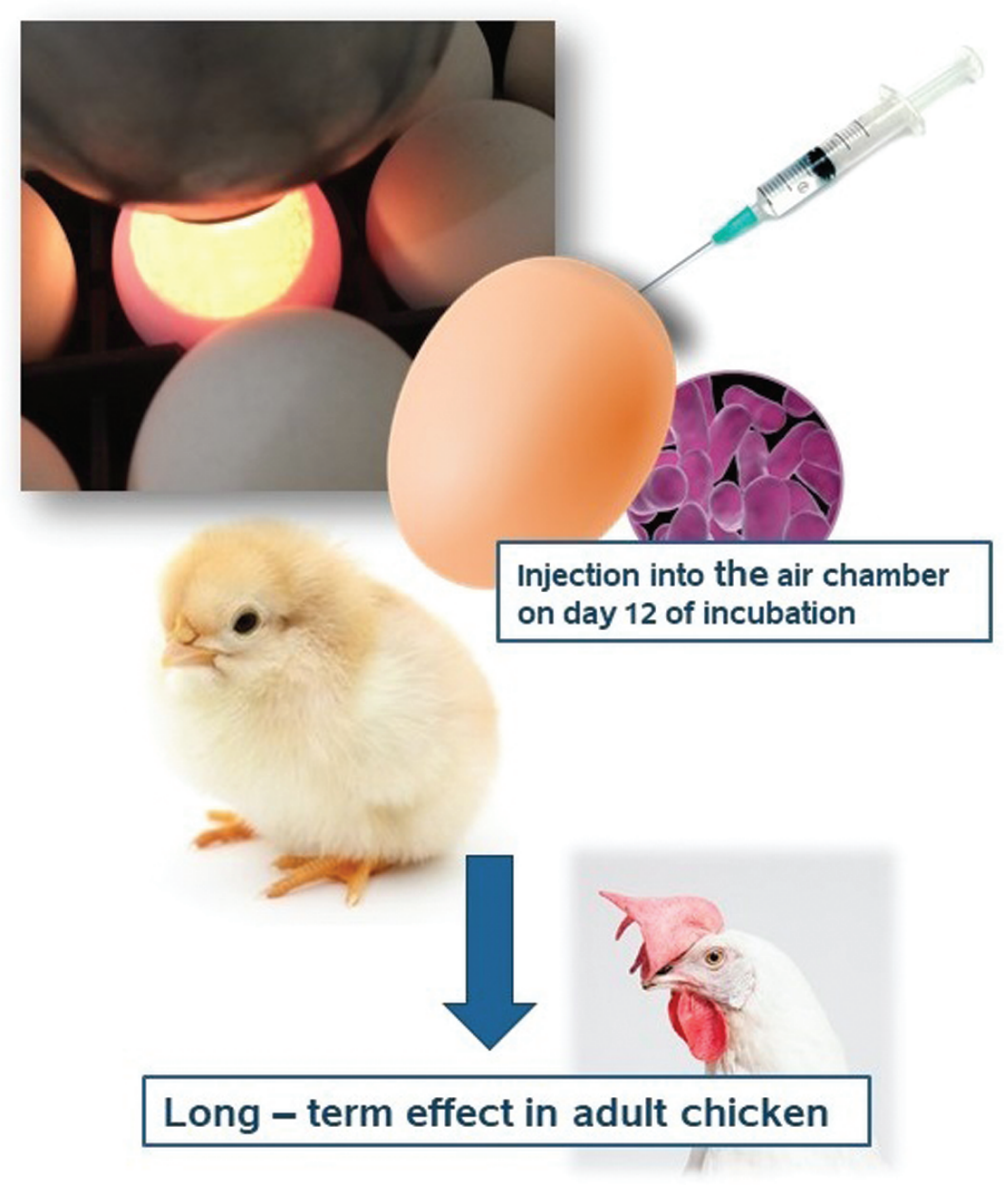
In ovo technology on day 12 of egg incubation for long-term effects throughout the rearing period.

The in ovo process is based on the delivery of a desired bioactive substance inside the egg containing the developing chicken embryo. The whole process is performed before hatching. It was originally designed for in ovo vaccination against Marek’s disease virus and bursal diseases in 18-d-old embryos. Later on, the process was adapted for in ovo feeding, which is a delivery of vitamins, carbohydrates, or proteins on day 18 of embryo development. Another alteration of in ovo technology is application of prebiotic/probiotic/synbiotic delivery on day 12 of egg incubation (Figure 2). The in ovo injection of 18-d-old embryo might be done into the amnion or the embryo itself. The in ovo delivery on day 12 of egg incubation is directed to the egg air cell. This injection site is safe for the developing embryo, which makes it easier for automatization. Yet another advantage of in ovo injection of a prebiotic/probiotic/synbiotic on day 12 of egg incubation is due to biological activities of these bioactive substances upon the injection and characteristics of the embryo’s environment inside the eggshell. At this point in embryo development, the chorioallantoic membrane is highly vascularized. Hence, the prebiotic deposited in the egg’s air cell is transferred into the circulatory system and further on to the developing intestine, while a probiotic injected into the egg’s air cell is available for the chicken embryo at the moment of breaking the inner membrane at the beginning of hatching. Therefore, this single in ovo injection on day 12 of egg incubation plays multiple roles for the developing embryo: to stimulate the endogenous microbiota (prebiotic), serve as a pioneer colonizer (probiotic), or both (synbiotic) (Siwek et al., 2018).

The impact of the in ovo administration of bioactives on the chicken embryo is lifelong. The changes, initiated by in ovo stimulation of the chicken gut microbiota, are determined long after hatching and are expressed in various phenotypic traits. From the perspective of poultry producers, the key parameters are: hatchability, chick mortality, and performance traits. It should be clearly noted that a properly optimized dose of bioactive for in ovo injection will have no harm on chick hatchability and will have a beneficial impact on the chicken gut microbiota. The positive impact on chicken gut microbiota is defined either by an increased number of indicatory bacteria eg, profile modification of *Bifidobacteria* in the upon the in ovo injection of raffinose family oligosaccharides (**RFO**) or galactooligosaccharides (**GOS**). A proper estimation of the impact of in ovo stimulation during embryo development on the production parameters requires large-scale experiments in industrial settings. In fact, our group has performed a large-scale trial including 275,000 broilers which received RFO in ovo on the day 12 of embryo development. This was a proof of concept, validating beneficial impact of prebiotics delivered in ovo on body weight, carcass weight, carcass yield, and breast muscle weight (Siwek et al., 2018).

The effects of the in ovo stimulation on the level of production traits are strictly dependent on the morphology, digestion, and absorption of the chicken intestines. Histological analysis of various parts of the chicken small intestine (duodenum and jejunum) is a key analysis of in ovo trials. In general, the in ovo administration of prebiotics or synbiotics increases the width and surface area of intestinal villi and deepens intestinal crypts. The particular effects are strictly related to the: bioactive used (prebiotics: inulin and GOS; synbiotics: *Lactobacillus salivarius* combined with GOS and *Lactobacillus plantarum* combined with RFO), time point of the analysis (day 1, 4, 21, or 42 post hatching), and part of the small intestine (duodenum and jejunum). Nevertheless, these changes increase the absorbing surface of the intestines. The administration of synbiotics in ovo has an impact on yet another important parameter of the jejunum and ileum, which is the number of goblet cells. Goblet cells produce mucus which creates a physical barrier in the guts (Siwek et al., 2018).

The changes initiated in the host gut microbiota upon administration of bioactives in ovo have a significant impact on the host GIT and entire chicken. From the perspective of the chicken broiler producers, one of the key traits is meat quality, a trait directly related to muscle histology. Prebiotic GOS delivery in ovo increased the level of lipid oxidation in the chicken meat during the storage time. The same prebiotic also led to increases in the intramuscular fat content and amount of polyunsaturated fatty acids of the breast muscle. Prebiotics were proved to have a positive impact on breast muscle weight and yield.

The effects of the prebiotics and synbiotics administration in ovo were also analyzed in the host immune system. The synbiotics delivered into the developing chicken embryo have an impact on the post-hatching development of gut-associated lymphoid tissue (**GALT**), high colonization of GALT by T cells in cecal tonsils, and enhanced B-cell proliferation in peripheral lymphatic organs (Siwek et al., 2018). Further study showed that early in ovo treatment of chicken embryos with prebiotics and synbiotics might not only have an impact at the structural level of the immune system but may also temporarily modulate production/maturation of leukocytes and their reactivity (Stefaniak et al., 2019).

All the above-mentioned phenotypic effects were detected in regular rearing conditions. Hence, we have also tested our in ovo approach on broiler chickens exposed to heat stress conditions. The prebiotic GOS injected in ovo on day 12 of egg incubation did mitigate negative effects of heat stress on performance, welfare, and meat quality traits of broiler chickens (Slawinska et al., 2019b; Tavaniello et al., 2020).

**Figure 3.**
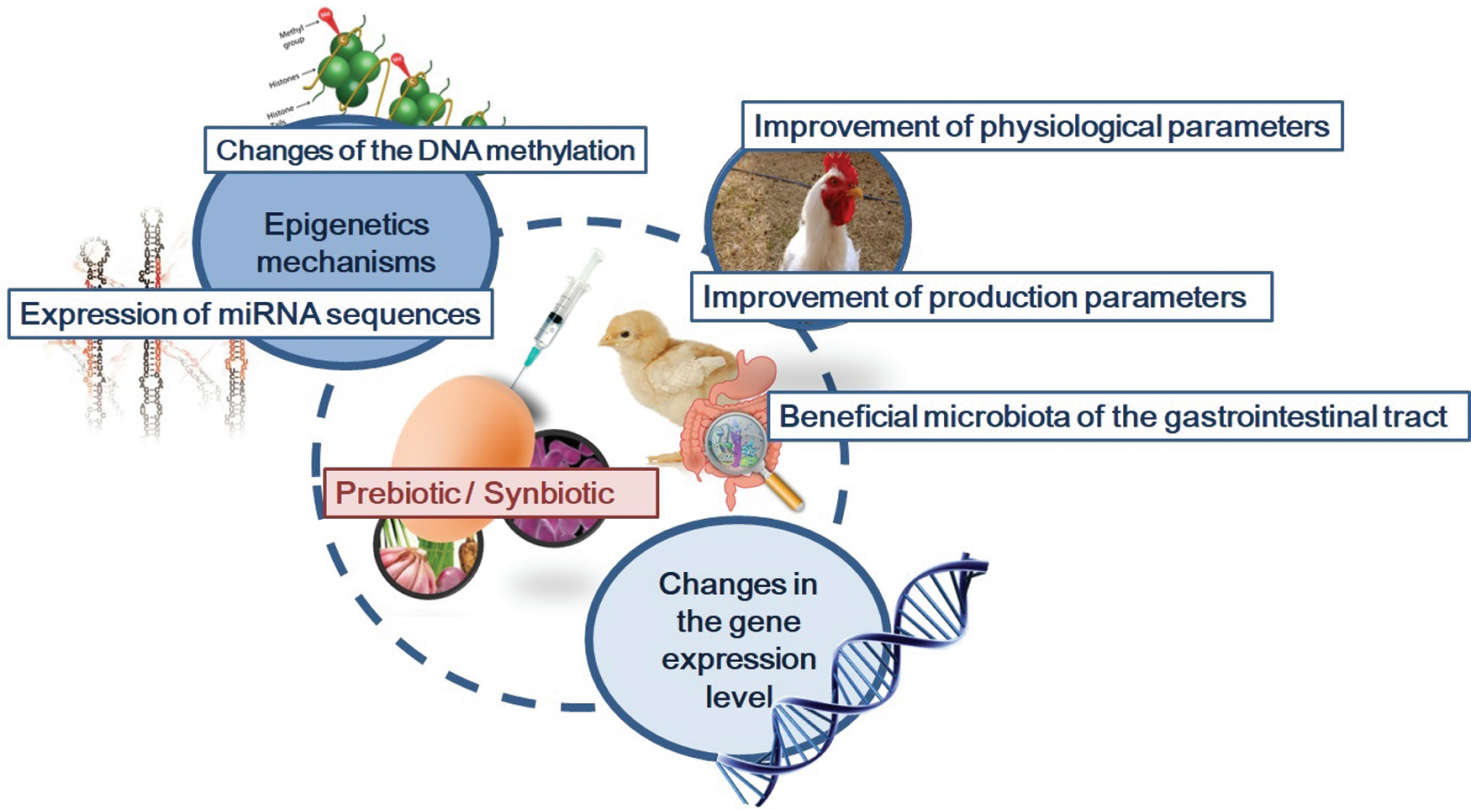
Molecular and phenotypic effects after in ovo administration on day 12 of egg incubation of prebiotics and synbiotics.

There are proposed four mechanisms behind the long-term effects of the prebiotics and synbiotics on the chicken organisms (Ajuwon, 2016). Three of them we might identify in relation to outcome of our studies (Dunislawska et al., 2017, 2021, 2020b). The first proposed mechanism is related to maintaining a normal intestinal microbiota by competitive exclusion and antagonism. We assume that this mechanism plays a very particular role in the case of in ovo delivery. This route of bioactives administration promotes the colonization of chicken GIT by beneficial microbiota. The second proposed mechanism is altering the metabolism by increasing digestive enzyme activity and decreasing bacterial enzyme activity and ammonia production. The third mechanism behind the long-term effects of prebiotics and probiotics is related to stimulation of the host immune system. In ovo stimulation affects the development of the immune system, including changes in the structure of the central and peripheral lymphatic organs (Madej et al., 2015; Madej and Bednarczyk, 2016). We also have recognized an impact of this mechanism in our earlier studies in the development of GALT, T-cell and B-cell proliferation, and modulation of gene expression in the immune organs (Sławińska et al., 2014; Madej et al., 2015; Slawinska et al., 2016). We also proved that the potency of immune stimulation differs among bioactives. Administration of a synbiotic provides a strong stimulus to the immune organs of growing chickens, while the strength of its stimulation depends on the genotype. In addition, in ovo stimulation influences the immune phenotype and cell distribution in cecal tonsils, ileum, and bursa of Fabricius of broiler chicken (Madej and Bednarczyk, 2016) (Figure 3). The fourth mechanism behind the prebiotics/probiotics administered in poultry is related to improved feed intake and digestion. The effects that are linked to this mechanism are changes induced by prebiotics and synbiotics in the histology of the intestines such as deepening intestinal crypts.

## Gene Silencing after In Ovo Stimulation

The impact of in ovo-delivered prebiotics and synbiotics on the level of gene expression post hatching is strictly dependent on the type of bioactive and the time point of the analysis. Nevertheless, the general picture shows that immune-related genes in cecal tonsils and spleen are downregulated upon bioactives administration in ovo (Siwek et al., 2018). Gene modulation upon synbiotic injection in ovo has also been detected in the liver and muscle tissue (Dunislawska et al., 2019, 2020a). We have shown that synbiotics act as regulator of not only gene transcription but also protein expression (Dunislawska et al., 2021). Gene expression silencing was identified in intestinal, immune, and metabolic tissues after in ovo delivery of a prebiotic, probiotic, and synbiotic (Slawinska et al., 2016, Dunislawska et al., 2019). The phenomenon of negative regulation of gene expression may be due to the stimulation of the intestinal microbiota during embryonic development. Silencing of the immune-related gene expression may be associated with redirecting metabolic energy to growth and development instead of supporting the stimulation of the immune system (Kominsky et al., 2010). This gene silencing can be related to epigenetic regulation of gene expression. We hypothesize that downregulation of gene expression might be dependent on epigenetic mechanisms (Figure 2). The hypothesis concerning the influence of the modified microbiota on epigenetic gene regulation after in ovo administration of GOS prebiotic, and also GOS-based synbiotics, is supported by changes in the bacterial profile in the ileum and cecum. The synbiotic reduced the total amount of microbiota in the ileum. This effect is beneficial because the density and activity of the microbiota should be minimized in the upper intestine (i.e., in the ileum) and increased in the lower segment (i.e., in the cecum). The cecum serves as the main fermentation chambers with the highest activity and density of anaerobic bacteria (Dunislawska et al., 2017). Relative analysis of the abundance of bacteria in the intestinal contents after GOS in ovo administration showed that the section of the intestine and the prebiotic treatment had a significant effect on the abundance of *Bifidobacterium* spp. and *Lactobacillus* spp. (Slawinska et al., 2019a).

**Figure 4.**
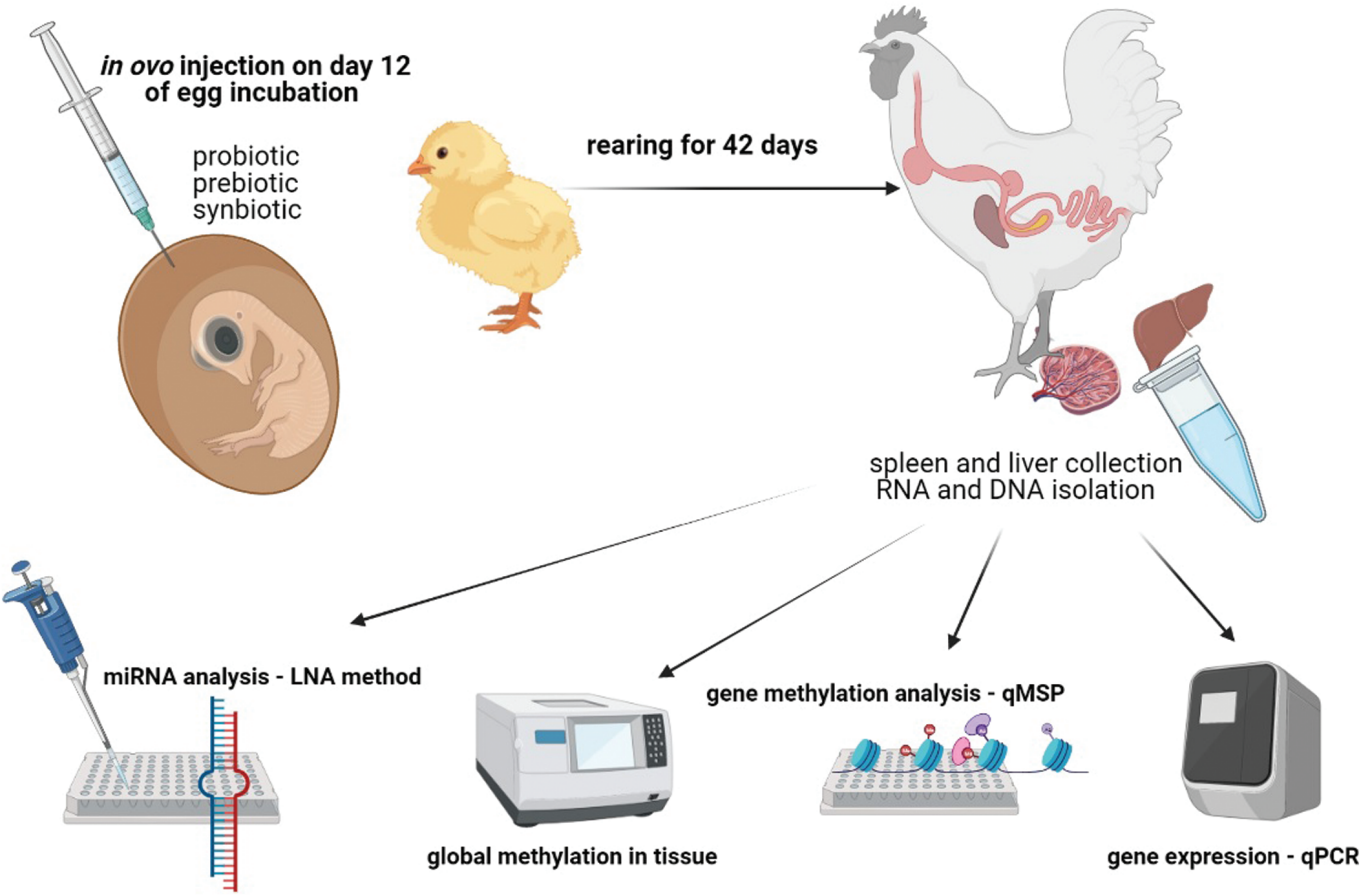
Experimental setup of epigenetic regulation analysis (global and gene methylation, miRNA expression) based on tissues collected from in ovo-stimulated chickens (created in BioRender.com).

Identifying the mechanisms driving gene silencing would be a key to understanding the molecular basis of environmental effects on phenotype (Ghavifekr Fakhr et al., 2013). Despite the well-known gut microbiome and the growing knowledge of epigenetic regulation, such as through DNA methylation, there is little research linking these two issues (Berghof et al., 2013). In experiments carried out by our teams, we assumed that the changes in gene expression induced by in ovo stimulation were dependent on epigenetic processes (Figure 4).

### Methylation level in immune and metabolic tissues

In the process of embryogenesis, just after the formation of the zygote, DNA demethylation occurs, and a new methylation profile begins to establish de novo (Gao et al., 2017). Due to this fact, the living environment and egg composition, or even the conditions of incubation of fertilized eggs, can significantly affect the methylation of the embryo’s DNA. The methylation process is influenced by many components found in poultry nutrition: selenium, folic acid, flavonoids, and probiotics (Jaenisch and Bird, 2003). Methylation is tissue specific. Still, little is known about tissue-specific DNA methylation and its potential causal role in shaping the immune response and supporting metabolic activity in poultry.

The fermentation product of the prebiotic tested (butyrate), which is an SCFA, may have a significant impact. In our research, we determined that bioactive substances delivered in ovo did not change the methylation pattern in the blood of adult broilers but influenced changes in the spleen. The results of the global methylation analysis indicate that the response after administration of the probiotic (*Lactococcus lactis* subsp. *cremoris*) is similar to the control group (where saline was administered). Prebiotic (GOS) and synbiotic are statistically significantly different from probiotic, without differentiating from each other. Administration of an exogenous bacterium (probiotic) in ovo is not an as strong environmental factor as a prebiotic or synbiotic. It can be assumed that in the symbiotic, it is the prebiotic component that plays a key role in modulating the gene expression profile and DNA methylation. In the DNA methylation analysis of individual genes in the chicken spleen (e.g., *NR4A3*, *IKZF1*, *NFATC1*, and *TNFRSF14*), the downregulation of mRNA abundance correlated with increase DNA methylation. In a subset of genes (e.g., *SYK* and *ANGPTL4*), this relationship has not been confirmed, which suggests the effect of the substance (but not epigenetic mechanism) on gene expression downregulation and the interference of other molecular mechanisms. Some genes have been hypomethylated, which suggests that bioactive substances delivered in ovo may also be associated with a reduction in methylation. Consequently, the bioactives also change gene expression patterns (Dunislawska et al., 2021).

Modulatory effects on mRNA expression and the DNA methylation profile after in ovo stimulation were analyzed in various genotypes, for example, broiler chicken and native chicken breed (Green-legged Partridge [**GP**]). As a result of intensive selection, the broiler chicken is characterized by high resistance and excellent production parameters, whereas GP is characterized primarily by low environmental and nutritional requirements. There is no selection within this breed of chicken, which may result in differentiation in response to stimulation of the intestinal microbiota compared with broiler chickens (Dunislawska et al., 2021).

Our comparison confirmed the significant influence of genotype on DNA methylation. In both genotypes, the effect of bioactive substances delivered in ovo on intestinal microbiota profiling was demonstrated (Dunislawska et al. 2020c, 2021)

Analysis of individual silenced genes in the liver showed that the synbiotic RFO combined with *L. plantarum* led to hypermethylation of *ANGPTL4* gene. This gene is responsible for the inhibition of lipoprotein lipase, which leads to the reduction of the fat storage. Even though chicken weight remained unchanged between experimental groups, changes in the fatty acid profile and lipid content were noted, which positively influenced the nutritional value of the meat (Dunislawska et al., 2020b). Methylation of *NR4A3* related to the regulation of fatty acid consumption and muscle mass decreased after administration of synbiotics in ovo. Gene expression was also negative after administration of a GOS-based synbiotic with *L. salivarius*. Other reports suggest that hypomethylation alone is insufficient in many cases to activate silenced genes. Administration of an RFO-based synbiotic showed strong hypermethylation of the gene with a concomitant decrease in gene expression in the liver (Dunislawska et al., 2020b).

### miRNA expression in the liver

miRNAs are the fraction of small RNA molecules encoded in the genome that have a fundamental impact on gene expression. Mature miRNA binds to the 3′-untranslated regions (UTRs) end of the regulated mRNA molecule of the target gene, destabilizing it and preventing translation. This way, miRNA affects targeted genes silencing (Taganov et al., 2007). There are reasons to link miRNA activity with modification of DNA methylation through interaction with newly formed mRNA strands of the target gene. The major methyltransferases in animals are believed to be regulated by miRNAs (Chuang and Jones, 2007). miRNA plays an important role as a component of the molecular machinery of host–probiotic interaction We confirmed modulatory role of bioactives delivered in ovo in the liver. In the liver of broiler and native chickens, all in ovo-delivered compounds (i.e., GOS prebiotic, *L. lactis* subsp. *cremoris* probiotic, and the synbiotic composed of both substances), the increased activity of miRNA was determined. We showed the activity of 3 miRNAs (in broiler chicken) and 6 mRNAs (in native chicken) out of 10 miRNAs. Interestingly, significant activation of the miRNAs in the chicken liver occurred after the administration of the probiotic and the synbiotic. It indicates that the probiotic component is responsible for the miRNA activity after administration of the synbiotic (Sikorska et al., 2021). Expression of miRNA after administration of the prebiotic decreased compared with the control, which suggests that the role of the prebiotic itself in the process of miRNA activity is negligible. Its potential lies in supporting the probiotic component in one synbiotic product (Sikorska et al., 2021). The literature shows that the probiotic can participate in the interaction between the microbiota and the host influencing miRNA expression (Teng et al., 2018). Scientific reports show that miRNAs play a key role in the host’s immune response. Increased expression of miRNAs responsible for alleviating inflammation was demonstrated after the administration of a probiotic containing the *L. plantarum* strain (Rodríguez-Nogales et al., 2018). Delivery of probiotic can also be effective in relieving inflammation in poultry that are infected with *Salmonella* (Chen et al., 2017).

## Transgenerational Effects

Compared with mammals, birds have several advantages for studying transgenerational epigenetic inheritance (Guerrero-Bosagna et al., 2018). Chickens show an early sexual maturity, a high rate of egg production (over 300 eggs/yr), and shorter interval between generations, as well as requiring small floor space and less feed. Moreover, laying chicken breeds with a short generation interval (average 2.5 generations a year) are especially attractive for carrying out long-term, multigenerational genetic studies. Furthermore, by using semen diluent and artificial insemination, a virtually unlimited number of offspring can be obtained from one rooster. One major advantage is that a bird’s embryo develops outside of the mother, and the maternal influence is reduced only to the egg composition. Other environmental factors, such as the temperature of incubation and humidity, could be strictly controlled to minimize interindividual environmental variability (Guerrero-Bosagna et al., 2018).

To better understand the potential of epigenetic mechanisms after bioactive substances administered in ovo, it is necessary to analyze the direct effects and their intergenerational and/or transgenerational inheritance (Bednarczyk et al., 2021). The epigenetic effects can be classified into two categories: the so-called “context-dependent” or “germline-dependent” (Burggren, 2015). Context-dependent epigenetic inheritance affects the phenotype through a direct and continuous exposure to an environmental stressor within or across generations, and the phenotype remains modified only in the presence of a stressor. By contrast, germline-dependent inheritance results when the germline of an organism is directly affected, and the consequent phenotypic modifications persist across generations in the absence of the original causative agent (i.e., the environmental stressor). As such, only the altered phenotypes occurring in the second (in the case of male transmission) or third (in the case of female transmission) generation after a trigger can truly be described as transgenerational.

Numerous studies (Bentz et al., 2016; Li et al., 2016; Vinoth et al., 2018) have demonstrated inter- or multi-generational effects of changing environment in birds and also different developmental epigenetic patterns have been studied in various chicken types (Bednarczyk et al., 2021). These studies have proved that the chicken transcriptome could be reprogrammed by manipulation of different environmental factors during early embryogenesis. So far, however, only Leroux et al. (2017) described evidence of a transgenerational inheritance phenomenon in a bird species, although it is yet unclear which mechanisms may be involved.

## Perspectives

Going forward, the research holds the potential to help us program gut microbiota during embryonic development in a way that leads to stable and heritable gene silencing. Knowledge of host–pathogen interactions will provide a better understanding of epigenetic changes that can be used to determine their role in shaping poultry health and productivity. Epigenetic regulation of gene expression related to early in ovo stimulation and programming of the gut microbiota at the embryonic stage requires further analysis, especially in terms of heritability of effects and testing of substances that influence this effect. Our current reports allow us to conclude that the administration of bioactive substances (prebiotic, synbiotic, and probiotic) in ovo on day 12 of egg incubation has the potential to program the intestinal microbiota during embryonic development and specifically silence gene expression through DNA methylation and miRNA activity. These results constitute a basis for the initiation of further research and conceptual work, especially in the field of gene expression control through the interaction of various substances on the intestinal microbiota.

## Summary

In this short review, we showed the line of research that started with in ovo inoculation of the incubating chicken egg with prebiotic solution and concluded with studying epigenetic effects of said treatment. The microbiota is a complex environment that leads to deep physiological and molecular changes in the host organism. In ovo stimulation is a powerful, yet underestimated, method to control the microbiota in poultry and introduce epigenetic modifications at different levels. Different bioactive compounds stimulate different sets of traits and different sets of genes in different genotypes. As a consequence, bioactive compounds allow for practically unlimited modulation of many desired traits in poultry, driven by the intestinal microbiota reprogramming. There are still many questions to be asked and answered, but in ovo stimulation in poultry is definitely an interesting path to pursue.

## About the Authors

**Aleksandra Dunislawska** is an Assistant Professor in the Department of Animal Biotechnology and Genetics, Faculty of Animal Breeding and Biology of UTP University of Science and Technology in Bydgoszcz, Poland. She obtained her PhD in Biotechnology from West Pomeranian University of Technology in Szczecin, Poland. She completed a research internship at Aarhus University (Denmark) in the Department of Molecular Biology and Genetics, Center of Quantitative Genetics and Genomics. She is leader of the researcher project entitled “Epigenetic regulation of gene expression in chickens after in ovo administration of a prebiotic and synbiotic” financed by the Polish National Science Center. Her research interests include host–microbiome interaction, programming of the intestinal microbiota using bioactive substances, functional analysis of the animal and human microbiome, epigenetic regulation of gene expression, omics analyses, and in vitro research models.

**Anna Slawinska** is an Associate Professor at Faculty of Animal Breeding and Biology of UTP University of Science and Technology in Bydgoszcz, Poland. She obtained her PhD in Animal Sciences from UTP University of Science and Technology in Bydgoszcz, Poland, and DSc in Biotechnology from Marie Curie University in Lublin, Poland. Her employment history includes Assistant Professor at UTP University of Science and Technology in Bydgoszcz, Poland; Consultant at FAO/IAEA Agriculture and Biotechnology Laboratory in Vienna, Austria; Fulbright Visiting Scholar at Iowa State University in Ames, USA; and Research Fellow at University of Molise in Campobasso, Italy. Her research interests include molecular biology, host-microbiota interactions in poultry, the concept of resilience, immunomodulation, and intestinal health, in vitro models, gene expression regulation, and microbiota as epigenetic cue in farm animals.

**Maria Siwek** is a full professor at the Animal Biotechnology and Genetics Department of UTP, University of Science and Technology, Bydgoszcz, Poland. She obtained her PhD at Wageningen University in the Netherlands. She obtained Marie Curie Fellowship twice. Her research interest is focused on structural and functional genomics in animal models. The most recent studies concern an early microbiota stimulation and its further impact on the host organism.

**Marek Bednarczyk** is a full professor and head of the Animal Biotechnology and Genetics Department at UTP University of Science and Technology in Bydgoszcz. He obtained his PhD in animal science at Poznan University of Life Sciences. His research focus is on in ovo chicken embryo manipulation.
